# Viral Etiology Associated With Acute Respiratory Tract Infection Patients in Bangkok, Thailand

**DOI:** 10.7759/cureus.66897

**Published:** 2024-08-14

**Authors:** Phattharaporn Inma, Nungruthai Suntronwong, Silpsiri Sinsulpsiri, Suriya Srimaneewiroon, Yong Poovorawan

**Affiliations:** 1 Center of Excellence in Clinical Virology, Faculty of Medicine, Chulalongkorn University, Bangkok, THA; 2 Center of Excellence In Clinical Virology, Faculty of Medicine, Chulalongkorn University, Bangkok, THA; 3 Bangpakok 9 International Laboratory, Bangpakok 9 International Hospital, Bangkok, THA

**Keywords:** prevalence, respiratory tract infection, thailand, etiology, virus, epidemiology

## Abstract

Background

Acute respiratory infections (ARIs) are a significant public health concern globally. After the relaxation of COVID-19 containment measures, there has been an increase in respiratory tract infections. However, the epidemiological data on circulating respiratory pathogens after the COVID-19 pandemic in Bangkok, Thailand are interesting. We conducted a study on the respiratory pathogens detected in patients with ARIs in 2023.

Methodology

This retrospective study utilizes archived nasopharyngeal swab samples from patients with ARIs of all ages at Bangpakok 9 Hospital between January and December 2023. These samples were collected when physicians ordered multiplex polymerase chain reaction testing as part of the diagnostic investigation. All samples were tested for 23 types of respiratory viruses and bacteria using multiplex reverse transcription-polymerase chain reaction (QIAstat-Dx, Qiagen) testing.

Results

Of 321 patients, viral pathogens were found in 78.5% of cases, with 21.5% remaining unidentified. Most patients (47%) were aged between two months to five years. The most common pathogen identified was rhino/enterovirus (28.0%), followed by human parainfluenza virus (15.1%), influenza virus (12.0%), respiratory syncytial virus (9.9%), human metapneumovirus (9.5%), adenovirus (9.2%), bocavirus (8.0%), and coronavirus (5.5%). Interestingly, the prevalence of SARS-CoV-2 infection was relatively low at 2.8%. Moreover, viral co-infection was observed in 25% of cases. Monthly distribution revealed the fluctuating prevalence of detected respiratory pathogens co-circulation throughout the year. In addition, the proportion of identified pathogens varied among patients across all age groups.

Conclusions

Our study reported the high prevalence of respiratory pathogens in ARI patients of all ages after the COVID-19 pandemic. The most prevalent respiratory pathogens among ARI cases were viruses, particularly rhino/enterovirus. The data hold significance for physician awareness regarding diagnosis, treatment, and the implementation of infection control strategies in cases of ARIs.

## Introduction

Acute respiratory infections (ARIs) have been considered a significant worldwide public health issue associated with high mortality and morbidity rates [1]. ARIs are commonly caused by viruses, including adenovirus (ADV), bocavirus virus (BoV), coronavirus (CoV), human metapneumovirus (HMPV), influenza virus, rhino/enterovirus (RV/EV), respiratory syncytial virus (RSV), human parainfluenza (PIV), and severe acute respiratory syndrome coronavirus 2 (SARS-CoV-2) [2]. Additionally, specific bacteria such as *Mycoplasma pneumoniae*, *Legionella pneumophilla*, and *Bordetella pertussis* can also cause respiratory illness, particularly in children, although less commonly [3]. However, relying solely on clinical symptoms cannot completely differentiate viral or bacterial respiratory infections. Laboratory diagnostics are crucial for identifying the causative pathogens which assists physicians in comprehending the epidemiology and clinical signs of circulating pathogens and is essential for infectious disease management.

Typically, viral pathogens commonly affect the upper respiratory tract causing mild symptoms. Some viral infections can cause severe symptoms of the lower respiratory tract infection such as bronchitis and pneumonia resulting in the fourth-highest global cause of mortality in 2019 [4].

In December 2019, the SARS-CoV-2 pandemic caused a significant public health burden worldwide [5]. The incidence of respiratory infection caused by other viruses and bacteria was significantly reduced during the COVID-19 pandemic due to public health measures [6]. After the relaxation of COVID-19 containment measures at the end of 2022, the number of ARI cases with a diagnosis of non-COVID-19 infection among patients seeking medical care increased, accounting for the greatest number of antibiotic prescriptions [7].

In Thailand, the epidemiological data of viral and bacterial respiratory infection was reported in children in 2019-2020 before and during the early COVID-19 pandemic period [8] and showed that the most common cause of respiratory infection was a viral pathogen. However, there was a great diversity in the prevalence of etiological pathogens based on the differences in countries, regions, demographic populations, and seasonality. In addition, the epidemiology data of circulating respiratory pathogens causing ARI after the COVID-19 pandemic has been limited.

To enhance the understanding of epidemiological data of respiratory pathogens following the COVID-19 pandemic, we investigated the respiratory pathogens causing ARIs in patients of all ages in Bangkok, Thailand, between January and December 2023 using multiplex real-time polymerase chain reaction (PCR) testing. These findings will provide data on the prevalence and characteristic patterns of respiratory pathogens circulating post-COVID-19 pandemic, which may help in determining the appropriate treatment and clinical management for ARI patients.

## Materials and methods

Study design

This retrospective study enrolled consecutive patients of all ages who were presented with ARIs at Bangpakok 9 Hospital between January and December 2023. These samples were collected when physicians ordered multiplex PCR testing as part of the diagnostic investigation. ARI was defined as a fever exceeding 37.5°C, along with respiratory symptoms such as headache, myalgia, fatigue, diarrhea, nausea/vomiting, loss of appetite, loss of smell or taste, or increased work of breathing, or the onset of symptoms within 21 days [9]. The samples detected using other methods and/or those collected from the same individuals with repetitive pathogen detection were excluded from this study. The study protocol was approved by the Institutional Review Board of the Faculty of Medicine, Chulalongkorn University (approval number: 0376/67) and was conducted under the Declaration of Helsinki and Good Clinical Practice principles. Inform consent was waived as all demographic data were anonymous, and the study received permission from the director of Bangpakok 9 International Hospital.

Laboratory testing

All nasopharyngeal swabs, which were collected from patients with ARIs and stored in 1 mL of universal transport medium (UTM), were retrospectively obtained for multiplex reverse transcription polymerase chain reaction (RT-PCR) testing. The UTM contained the following ingredients: Hank’s balance salt, bovine serum albumin, L-cysteine, gelatin, sucrose, L-glutamic acid, HEPES buffer, vancomycin, amphotericin B, colistin, and phenol red.

Samples were tested using the QIAstat-Dx Respiratory SARS-CoV-2 panel (QIAGEN, Hilden, Germany) which is a more recent qualitative test panel designed to detect 23 viral and bacterial targets using multiplex RT-PCR testing [10]. In brief, 300 µL of the sample was transferred manually into the QIAstat-Dx Respiratory SARS-CoV-2 panel cartridge and tested on the QIAstat-Dx analyzer 1.0 [11].

This multiplex RT-PCR testing was designed for 23 respiratory pathogen targets including influenza A virus, influenza A subtype (H1N1/2009, seasonal H1N1, and seasonal H3N2), influenza B virus, common CoV (229E, HKU1, NL63, and OC43), PIV 1-4, RSV A/ B, HMPV A/B, ADV, BoV, RV/EV, SARS-CoV-2, *Mycoplasma pneumoniae*, *Chlamydophila pneumoniae*, *Legionella pneumophila*, and *Bordetella pertussis*.

Statistical analysis­­­­

The sample size was calculated using Cochran’s sample size formula, as previously described [12]. The parameters included the expected prevalence of respiratory pathogens from a previous study (68.9%) [13], a precision of 5%, and the Z statistic for a 95% confidence level (1.96). Descriptive data were presented as frequency and percentages of positive cases (defined as the number of positive samples among all tested samples). The percentage of positive tested samples for respiratory pathogens was determined for each month and plotted over time to observe the seasonal trends. We classified patients into the following different age groups: <5, 5 to <10, 10 to <20, 20 to <60, and ≥60 years. The proportion of different types of pathogens was determined in each age group. All analysis was conducted using SPSS Statistics version 21.0 (IBM Corp., Armonk, NY, USA).

## Results

For 321 patients (in and outpatients) with acute respiratory tract infections, nasopharyngeal swabs were collected at Bangpakok 9 International Hospital. There were 161 (50.2%) males and 160 (49.8%) females. The median age was five years (ranging from 2 months to 93 years). Most patients (47.4%) were aged between two months and five years.

Respiratory pathogen detection in acute respiratory infection patients

**Figure 1:**
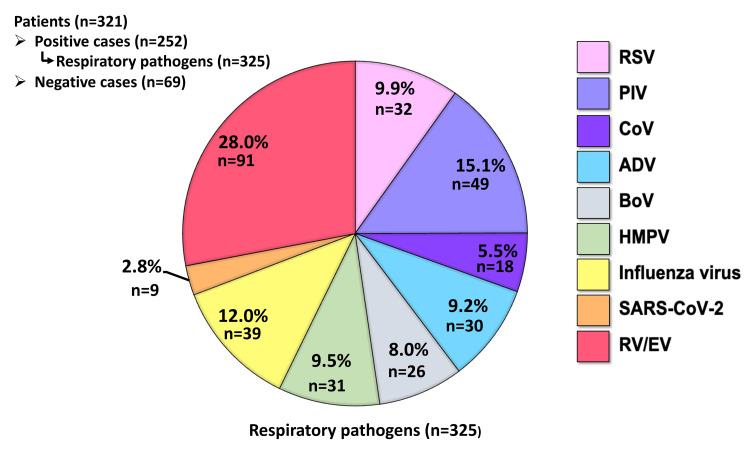
Proportion of respiratory pathogens detected in ARI patients. The pie chart illustrates the distribution of respiratory pathogens (n = 325) identified from the positive cases (n = 252) among ARI patients. The percentages represent the proportions of detected respiratory pathogens, each depicted in different colors. N indicates the number of identified respiratory pathogens in ARI cases of single and mixed infection (n = 325). ARI: acute respiratory infection; RSV: respiratory syncytial virus; PIV: human parainfluenza virus; CoV: coronavirus; ADV: adenovirus; BoV: bocavirus; HMPV: human metapneumovirus; SARS-CoV-2: severe acute respiratory syndrome coronavirus 2; RV/EV: rhino/enterovirus

From January to December 2023, of a total of 321 patients with ARIs, 252 (78.5%) cases revealed at least one respiratory pathogen, while the specific pathogen remained unidentified in 69 (21.5%) cases. Among the positive samples, the most common pathogen identified was RV/EV (28.0%), followed by PIV (15.1%), influenza virus (12.0%), RSV (9.9%), HMPV (9.5%), ADV (9.2%), BoV (8.0%), and CoV (5.5%), as shown in Figure 1. Interestingly, the prevalence of SARS-CoV-2 infection was relatively low at 2.8%.

Of the 252 positive cases, 63 (25%) were co-infected with two or more respiratory pathogens, leading to a total detection of 325 viral pathogens (Table 1). Among the co-infections with two viral pathogens (53/63), human PIV combined with RV/EV (8/53) and ADV combined with RV/EV (8/53) were the most common patterns of mixed infection. While co-infection with three viral pathogens (10/63) exhibited a more varied pattern. However, no bacterial infection was observed.

**Table 1:** Respiratory pathogen patterns identified in ARI patients with mixed infection. ARI: acute respiratory infection; RSV: respiratory syncytial virus; Flu: influenza virus; PIV: parainfluenza virus; CoV: coronavirus; ADV: adenovirus; BoV: bocavirus; HMPV: human metapneumovirus; SARS-CoV-2: severe acute respiratory syndrome coronavirus 2; RV/EV: rhino/enterovirus

Viral infection patterns	Number of samples
Two viral infections (n = 53)	
ADV + PIV	2
ADV + RSV	2
ADV + RV/EV	8
BoV + ADV	1
BoV + CoV	1
BoV + PIV	3
BoV + RSV	3
BoV + RV/EV	7
CoV + Flu	2
CoV + HMPV	4
CoV + SARS-CoV-2	1
Flu + RSV	1
Flu + RV/EV	3
Flu + SARS-CoV-2	1
HMPV + RV/EV	5
PIV + RV/EV	8
RV/EV + RSV	1
Three viral infection (n = 10)	
ADV + RV/EV + PIV	1
BoV + Flu + PIV	1
BoV + PIV + RV/EV	1
BoV + RV/EV + RSV	1
BoV + RV/EV + SARS-CoV-2	1
HMPV + RV/EV + CoV	1
PIV + Flu + RSV	1
PIV + HMPV + RV/EV	1
RSV + RV/EV + CoV	1
RV/EV + BoV + SARS-CoV-2	1
Total mixed infections	63

Seasonal distribution of respiratory pathogens

**Figure 2:**
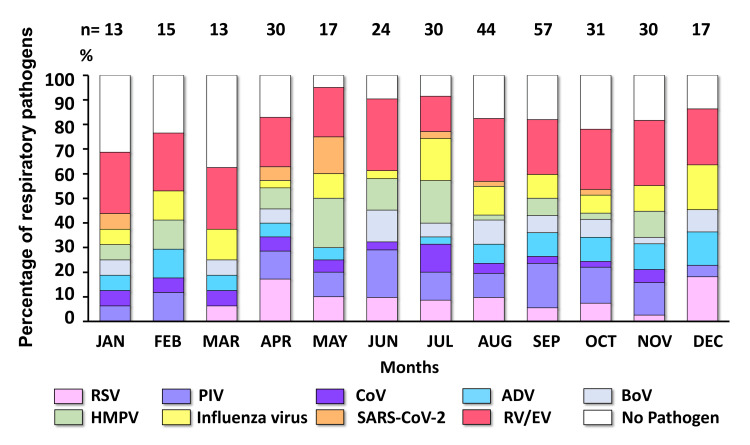
Monthly distribution of respiratory pathogens detected in ARI patients (n = 321) between January and December 2023, in Bangkok, Thailand. The stacked graph illustrates the proportion of each respiratory pathogen identified from positive cases and cases with no pathogen detection, which are displayed in different colors. The number above bar graph indicates the total number of ARI patients collected in each month. ARI: acute respiratory infection; RV/EV: rhino/enterovirus; SARS-CoV-2: severe acute respiratory syndrome coronavirus 2; HMPV: human metapneumovirus; BoV: bocavirus; ADV: adenovirus; CoV: coronavirus; PIV: parainfluenza virus; RSV: respiratory syncytial virus

Overall, the monthly detection rate of respiratory pathogens ranged from 62.5% to 91.4% during the study period. The highest number of positive cases were found between June and October (rainy season), accounting for 57.9% (186/321), followed by 23.4% (75/321) and 18.7% (60/321) observed between November and February (winter season) and between March to May (summer season), respectively (Figure 2). Among the positive samples detected in ARI patients, the distribution of viral pathogens showed seasonal diversity and was co-circulated throughout the calendar year. Our results showed that RV/EV circulated year-round and exhibited the highest monthly prevalence among viral pathogens, with proportions ranging from 14.3% to 29% during the study period. The positive rates of PIV and BoV increased during the rainy season, peaking in June at 19.4% and 12.9%, respectively. Although the influenza virus circulated year-round, two peaks were observed between July and September (9.7-17.14%) and a second peak in December (18.2%). Additionally, RSV was detected from April to December, with peaks appearing in April (17.1%) and December (18.2%). HMPV ascended between May and July with a monthly detection rate exceeding 10%. ADV slightly increased during the winter season and hit a peak in December. Besides, the monthly positive detection of CoV remained relatively stable, mostly staying below 6% throughout the year.

Prevalence of respiratory pathogen infection by age groups

To demonstrate the age-specific distribution of respiratory pathogens, ARI patients were categorized into five different age groups, including <5 (n = 152, 47.3%), 5 to <10 (n = 111, 34.6%), 10 to <20 (n = 33, 10.3%), 20 to <60 (n = 15, 4.7%), and ≥60 (n = 10, 3.1%) years old. Our results showed that 81.6% of patients aged <5 years (124/152), 73.9% of those aged between 5 and <10 (82/111), 81.8% of those aged between 10 and <20 (27/33), 73.3% of those aged between 20 and <60 (11/15), and 80% of those aged ≥60 years old (8/10) tested positive for respiratory pathogens.

**Figure 3:**
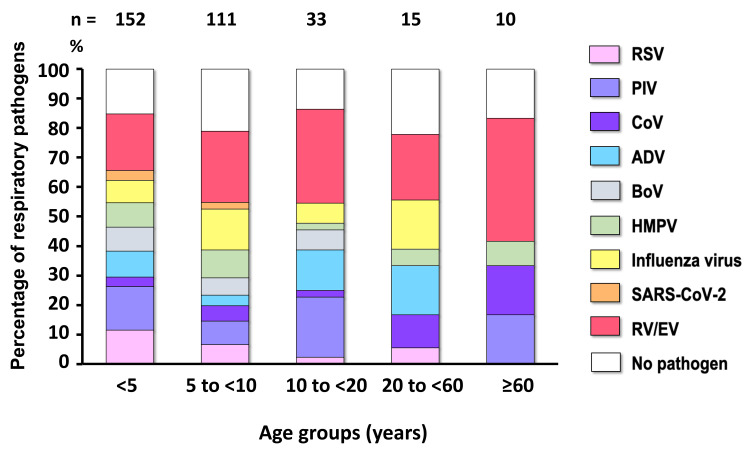
The prevalence of viral respiratory infection detected in ARI patients (n = 321) by age groups. The stacked graph illustrates the proportion of each respiratory pathogen identified from positive cases and cases with no pathogen detection, which are displayed in different colors. The number above the bar graph indicates the total cases of ARI patients per age group. ARI: acute respiratory infection; RV/EV: rhino/enterovirus; SARS-CoV-2: severe acute respiratory syndrome coronavirus 2; HMPV: human metapneumovirus; BoV: bocavirus; ADV: adenovirus; CoV: coronavirus; PIV: parainfluenza virus; RSV: respiratory syncytial virus

The proportion of viral pathogens responsible for causing ARIs in each different age group is shown in Figure 3. Notably, RV/EV accounted for the majority of viral pathogens detected in all age groups of patients. In addition to RV/EV, RSV (11.5%) and PIV (14.8%) were mostly distributed to cause infections in patients aged <5 years old. Whereas the influenza virus (13.9%) was prevalent in causing infection in the 5 to <10 age group. Additionally, individuals in the 10 to <20 age group were more likely to be infected by PIV (20.5%) and ADV (13.7%). Moreover, patients in the 20 to <60 age group were mostly detected with influenza virus (16.7%) and ADV (16.7%). Whereas PIV (16.7%) and CoV (16.7%) were more likely to cause infection in patients aged ≥60 years old. Therefore, the age-specific characteristics of respiratory pathogens showed that RSV was mostly detected in children <5 years old but gradually decreased in prevalence with age. While influenza, RV/EV, and PIV were typically found in both children and adults. Furthermore, ADV and CoV were most prevalent in causing infection in adults.

## Discussion

In this study, we demonstrated the prevalence of respiratory pathogens in ARI patients following the major outbreak of COVID-19 in Bangkok, Thailand, in 2023. Our findings report an overall positive rate of 78.5% for 23 types of multiplex RT-PCR testing. This result reveals that the majority of ARIs in patients are caused by viruses. Notably, the variation of detected respiratory viruses was observed across all age groups. RV/EV was the most detected virus during the study period, followed by PIV, influenza, and RSV. However, we found that only 21.5% of ARI cases were caused by unidentified respiratory pathogens. This result might occur due to a lack of detection of common pathogens in this method such as *Streptococcus* and *Staphylococcus* [14].

Several pathogens were the prevalent causes of respiratory infections before the COVID-19 pandemic. Picornaviruses, including rhinoviruses, caused 22.5% to 32.9% of cases. Influenza infected 22.5% to 28.7% of patients. RSV affected 11.1% to 15.9% of patients [8,15]. Bacteria were less frequently identified as causes of respiratory infections [14]. During the COVID-19 pandemic, a meta-analysis indicated that the prevalence of most respiratory pathogens, including RSV, influenza, PIV, and ADV, decreased compared to pre-COVID-19 [16]. SARS-CoV-2 was the dominant respiratory pathogen during the pandemic. In this study, the detection rate of SARS-CoV-2 was 2.8%, and the incidence of SARS-CoV-2 was quite low. This result might be related to most patients with respiratory illnesses tested for the virus themselves using antigen test kits (ATKs). Once they knew the results, they either treated themselves or did not test for other respiratory pathogens. Therefore, the incidence of SARS-CoV-2 is likely lower or underestimated.

Our study found that RV/EV was the most prevalent respiratory virus, which aligns with a previous study that reported the dynamic pattern of respiratory pathogens post-COVID-19 in Saudi Arabia [17]. Agca et al. [13] reported that while rhinovirus infections continue to be the leading cause of colds, comprising more than 50% of such illnesses, our hospital did not observe a significant impact of SARS-CoV-2 on rhinovirus activity levels. Our observations suggest that the COVID-19 pandemic may not have significantly impacted RV/EV activity.

In Thailand, ARIs occur throughout the year, peaking during the rainy season or the beginning of the school term, especially from June onwards. Influenza activity peaked twice between February and March and again between August and September [18]. RSV season typically spans from July to November each year, differing from regions in the northern and southern hemispheres where respiratory illnesses are more common during the winter season [19]. However, our study showed a seasonal diversity of respiratory pathogens which fluctuated and co-circulated throughout the year.

Common viruses found in ARI patients included RV/EV, comprising rhinovirus A, B, and C, as well as enterovirus D 68 and EVD 68, followed by RSV, PIV, and Influenza viruses [20,21]. Our findings highlight the prevalence of respiratory viruses across different age groups. RV/EV is one of the most detected pathogens across various age groups, followed by PIV and influenza virus, while RSV was most prevalent in children. These results were supported by previous studies showing that RV/EV is most prevalent in both children and adults, while RSV is more common in infants and older adults. Further, influenza is frequently found in children aged six and above, as well as in adults. HMPV can be found in all age groups [8,19,22].

There are several limitations of this study. Initially, the number of ARI cases was relatively small and most participants were children aged <5 years. This limitation may make it difficult to discern age and seasonal patterns of viral infections. However, its strength lies in the diverse range of viruses studied simultaneously. In addition, the number of SARS-CoV-2-positive cases was low in this study and only detected in children. This limitation may be related to patients who tested for SARS-CoV-2 using ATKs and not seeking hospital care due to mild or asymptomatic cases. In case of testing positive or infected, they were not undergoing RT-PCR testing. Moreover, the test panel used in this study included only four bacteria, and we did not conduct direct culture tests for other bacteria that can cause ARIs such as *Burkholderia pseudomallei*, *Corynebacterium diphtheriae*, *Haemophilus influenzae*, and *Streptococcus pneumoniae*.

## Conclusions

Our study reported a high prevalence of respiratory pathogens in ARI patients of all ages after the COVID-19 pandemic. Our findings showed that most ARI cases are caused by viruses, with common ones including RV/EV, RSV, PIV, and influenza viruses. RV/EV was the most prevalent throughout the calendar year among ARI patients. Viral respiratory patterns vary across age groups and seasons. This information may assist physicians and authorities in developing appropriate prevention strategies for primary care settings, emphasizing minimal antibiotic use for ARIs, and considering appropriate antiviral treatment for patients diagnosed with influenza or COVID-19 (SARS-CoV-2).
